# Spoligotyping and *Mycobacterium tuberculosis*

**DOI:** 10.3201/1108.040982

**Published:** 2005-08

**Authors:** Andrea Gori, Alessandra Bandera, Giulia Marchetti, Anna Degli Esposti, Lidia Catozzi, Gian Piero Nardi, Lidia Gazzola, Giulio Ferrario, Jan D.A. van Embden, Dick van Soolingen, Mauro Moroni, Fabio Franzetti

**Affiliations:** * University of Milan, Milan, Italy;; † National Institute of Public Health and Environmental Protection, Bilthoven, the Netherlands

**Keywords:** Bacterial Typing, molecular epidemiology, multidrug resistance, tuberculosis

## Abstract

We evaluated the clinical usefulness of spoligotyping, a polymerase chain reaction–based method for simultaneous detection and typing of *Mycobacterium tuberculosis* strains, with acid-fast bacilli–positive slides from clinical specimens or mycobacterial cultures. Overall sensitivity and specificity were 97% and 95% for the detection of *M. tuberculosis* and 98% and 96% when used with clinical specimens. Laboratory turnaround time of spoligotyping was less than that for culture identification by a median of 20 days. In comparison with IS*6110*-based restriction fragment length polymorphism typing, spoligotyping overestimated the number of isolates with identical DNA fingerprints by ≈50%, but showed a 100% negative predictive value. Spoligotyping resulted in the modification of ongoing antimycobacterial treatment in 40 cases and appropriate therapy in the absence of cultures in 11 cases. The rapidity of this method in detection and typing could make it useful in the management of tuberculosis in a clinical setting.

## Abstract 2

Speed of spoligotyping could be a benefit in the clinical setting.

The last decade has seen a dramatic resurgence in the incidence of tuberculosis throughout the world and an increased need for more rapid methods to diagnose and prevent dissemination of this disease (1). Well-equipped clinical laboratories can detect tuberculosis cases within 14 to 21 days by using liquid culturing systems such as BACTEC (Becton Dickinson, Sparks, MD, USA). Moreover, several studies have verified the usefulness of nucleic acid amplification–based methods for diagnosis of *Mycobacterium tuberculosis* infections in <24 hours (2–4). Concomitantly, recently characterized molecular markers for typing mycobacterial strains have greatly facilitated and improved the study of tuberculosis epidemiology (5–8).

Restriction fragment length polymorphism (RFLP) typing with insertion element IS*6110* as a probe has become the most widely used method for differentiating strains of *M. tuberculosis* isolates (7,8). However, because the application of RFLP typing is restricted to mycobacterial cultures, 20–40 days are required before sufficient mycobacteria are available to obtain sufficient DNA needed for this method. This time restriction limits the usefulness of RFLP typing, especially in studying possible nosocomial transmission of tuberculosis in a clinical setting.

Spoligotyping, a new method for simultaneous detection and typing *of M. tuberculosis* complex bacteria, has been recently developed (9–11). This method is based on polymerase chain reaction (PCR) amplification of a highly polymorphic direct repeat locus in the *M. tuberculosis* genome. Results can be obtained from a *M. tuberculosis* culture within 1 day. Thus, the clinical usefulness of spoligotyping is determined by its rapidity, both in detecting causative bacteria and in providing epidemiologic information on strain identities. Implementing such a method in clinic settings would be useful in surveillance of tuberculosis transmission and in interventions to prevent further spread of this disease.

The aims of this study were to evaluate 1) the reliability of spoligotyping when used with clinical specimens, 2) the potential usefulness of the method in distinguishing *M. tuberculosis* from other nontuberculous mycobacteria (primarily *M. avium*), and 3) the feasibility and impact of spoligotyping in managing tuberculosis in clinical settings.

## Patients and Methods

### Specimen Collection

We conducted a 2-year survey of suspected cases of tuberculosis with spoligotyping of acid-fast bacilli (AFB)–positive specimens collected consecutively from January 2000 to December 2001 in the Microbiology Laboratory at L. Sacco Hospital in Milan. Three types of specimens were included. The first was material scraped from slides prepared from all Ziehl-Neelsen–positive clinical specimens, which were obtained from patients admitted to the hospital. These clinical specimens included sputum, bronchoalveolar lavage, bone marrow aspirate, feces, cerebrospinal fluid, and urine. The second was samples obtained from liquid culture medium containing growing mycobacteria (BACTEC, Becton Dickinson). The third was a mycobacterial colony grown on solid medium (Lowenstein-Jensen). The hospital microbiology laboratory conducted isolation (both in solid and liquid media), identification, and antimicrobial susceptibility testing by using standard methods (12) on all specimens. The results of spoligotyping were immediately provided to the physicians treating the patients.

### Clinical Characteristics

Demographic and epidemiologic data were obtained from the medical records of all patients with AFB-positive specimens, including medical history of mycobacteriosis, HIV status, dates and results of mycobacterial smears, signs, symptoms, radiographs of patients with tuberculosis, and CD4+ cell counts (for HIV-infected patients). Data regarding the response time of the method used, possible variation in treatments following spoligotyping results, and patient clinical responses were also obtained.

### Isolation of DNA

DNA was isolated from AFB-positive slides as previously described (13). Briefly, stained microscopic preparations were washed in xylol and absolute ethanol, scraped with a sterile blade, and collected in a microcentrifuge tube in 1× phosphate buffer. The samples were centrifuged for 10 min at 13,000 rpm. The pellets were resuspended in 100 μL lysis buffer (10 mmol/L Tris-HCl, 50 mmol/L KCl, 2.5 mmol/L MgCl_2_, 0.45% Tween 20, 0.45% Nonidet P40, and 10 mg/mL proteinase K) and incubated for 3 h at 56°C or overnight at 37°C. The samples were then incubated for 15 min at 95°C and centrifuged for 15 min at 13,000 rpm, and the supernatants were transferred to a new microcentrifuge tube and used in PCR. Mycobacteria were grown in culture, and their DNA was isolated as previously described (6).

### RFLP Fingerprinting Analysis and Spoligotyping

Isolates of *M. tuberculosis* were genotyped by RFLP using the IS*6110* probe as a genetic marker, as previously described by van Embden et al. (6). Spoligotyping was performed on genomic DNA by using the standard method described by Kamerbeek et al. (9). Control samples were used in these procedures as previously described (14).

### Computer-assisted Analysis of Typing Patterns

Gel Compar software version 4.1 (Applied Maths, Kortrijk, Belgium) was used to compare the hybridization patterns obtained by spoligotyping and RFLP fingerprinting. The software clustered strains with the same genotypic pattern and defined similarity dendrograms joining the obtained clusters. The results of the analysis were compared with our database containing all DNA patterns derived from tuberculosis cases analyzed (the database contains RFLP data from >4,500 different isolates in our region).

## Statistical Analysis

The sensitivity and specificity of spoligotyping in distinguishing *M. tuberculosis* from nontuberculous mycobacteria were calculated in comparison with culture results that excluded analysis of patients without culture confirmation. The sensitivity and specificity of spoligotyping in typing *M. tuberculosis* isolates were calculated in comparison with IS*6110* clustering.

## Results

### Patient Characteristics

Three hundred fifty AFB-positive slides from 164 episodes of suspected mycobacteriosis in 148 patients were analyzed. One hundred seven slides were obtained from fresh material: sputum (n = 65), stool (n = 19), lymph node aspirate (n = 12), bronchoaspirate (n = 4), urine (n = 3), skin biopsy (n = 2), biliar liquid (n =1), and pericardial fluid (n = 1). One hundred five samples were obtained after growth of mycobacteria from liquid medium, and 138 samples derived from culture of different materials (mainly blood, but also sputum, bone marrow aspirate, cerebral spinal fluid, and others) were obtained after growth on solid medium. The characteristics of the 148 patients are shown in Table 1.

**Table 1:** Characteristics of 148 patients with acid-fast bacilli in biologic specimens*

Age, y	
Median (range)	34 (3–88)
Mean	39.3
Sex, no. (%)	
Male	105 (70.9)
Female	43 (29.1)
Type of patient, no. (%)	
Infectious diseases	124 (83.8)
Pneumology	10 (6.8)
Internal medicine	10 (6.8)
Other	4 (2.7)
HIV status, no. (%)	
Negative	52 (35.1)
Positive	96 (64.9)
CD4+ cell count/μL†	
Median	47.5
Mean (range)	105 (1–589)
Previous tuberculosis, no. (%)	20 (13.5)
Previous MAC infection, no. (%)	10 (6.8)
No tuberculosis or MAC infection, no. (%)	118 (79.7)

*MAC, *Mycobacterium avium* complex. †Data available for 90 of 96 HIV-infected patients.

### Sensitivity and Specificity of Spoligotyping versus Culture

Culture confirmation was obtained in 317 (90.6%) of 350 AFB-positive slides from 138 of 164 episodes of suspected mycobacteriosis (Table 2). *M. tuberculosis* was isolated from 188 specimens from 77 patient episodes. Among these, isoniazid resistance was detected in 12 patients (15.6%), rifampin resistance in 11 patients (14.3%), streptomycin resistance in 4 patients (5.2%), and ethambutol resistance in 2 patients (2.6%). We also observed 6 patients with multidrug-resistant tuberculosis (resistance to at least isoniazid and rifampin). Fifty-six patients (72.7%) were infected with a strain susceptible to all 4 drugs.

**Table 2:** Comparison between spoligotyping and culture results in 350 acid-fast bacilli–positive samples

Mycobacteria grown in culture	No. episodes	Results of spoligotyping from clinical samples		Results of spoligotyping from liquid medium		Results of spoligotyping from solid medium		Total	
		Positive	Negative	Positive	Negative	Positive	Negative	Positive	Negative
*M. tuberculosis*	77	54	1	53	5	75	0	182	6
*M. avium*	28	0	15	0	20	2	31	2	66
*M. gordonae*	15	0	0	0	12	0	14	0	26
*M. xenopi*	8	0	2	1	5	1	6	2	13
*M. kansasii*	2	0	1	0	0	0	2	0	3
*M. chelonae*	2*	0	2	0	2	0	2	0	6
Other	6†	1	1	0	4	1	4	2	9
No growth	26	9	21	1	2	0	0	10	23
Subtotal		64	43	55	50	79	59	198	152
Total	164‡	107		105		138		350	
Sensitivity, %§		98.2 (71.1–98.4)		91.4 (75.7–90)		100		96.8 (86.2–97)	
Specificity, %§		95.5 (67.7–97.7)		97.7 (95.6–97.8)		93.6		95.3 (88.4–96.1)	

*Automated DNA sequencing rather than culture growth of *M. chelonae* confirmed one of these cases. †Other, 1 *M. fortuitum*, 1 *M. asiaticum*, and 4 nontyped mycobacteria. ‡16 patients had ≥2 episodes of mycobacterial infections. §Sensitivity and specificity were calculated without including the culture-negative specimens. Values in parentheses are ranges that include specimens with no growth.

One hundred ninety-eight of 350 AFB slides showed positive results by spoligotyping. Culture results confirmed the diagnosis of tuberculosis according to spoligotyping positivity in 182 (96.8%) of these 198 specimens. We did not observe definitive growth of mycobacteria in cultures from 10 patients. The PCR products obtained from 6 specimens of nontuberculous mycobacteria (2 *M. xenopi*, 2 *M. fortuitum*, and 2 *M. avium*) hybridized with *M. tuberculosis*–specific oligonucleotides, which indicated that that false-positive spoligotyping results were possible. However, mixed infections with 2 different mycobacteria cannot be ruled out.

The spoligotyping response was negative in specimens from 152 patients: 23 specimens showed no growth in culture, 123 were nontuberculous mycobacteria, and 6 showed growth characteristic of *M. tuberculosis* (Table 2). Three of these 6 false-negative spoligotyping results were from slides with very high concentrations of AFB, and a positive result was obtained when we repeated the test at a higher dilution (1:10).

In comparison with culture results, the sensitivity of spoligotyping was 98% for clinical specimens, 91% for slides obtained from liquid medium, and 100% for slides obtained directly from a mycobacterial colony on Lowenstein-Jensen solid medium. The corresponding specificities were 96% (clinical specimens), 98% (liquid medium), and 94% (solid medium), respectively (Table 2).

### Clinical Application of Spoligotyping

Under optimal conditions, spoligotyping requires <24 hours for results. However, in the present study, response time was evaluated, taking into consideration routine processing time in the laboratory. The time from receipt of clinical specimens to obtaining spoligotyping results was 1–26 days (median 6 days). However, spoligotyping results from clinical specimens were obtained a median of 20 days (mean ± SD, 22.9 ± 18.6) sooner than those obtained by culture confirmation of tuberculosis and a median of 29 days (35.0 ± 25.2) sooner than those obtained by susceptibility testing. In contrast, RFLP typing results were obtained after a median of 75 days (range 24–160) (Table 3).

**Table 3:** Time required for obtaining results with clinical specimens by spoligotyping compared with 3 other methods*

Procedure	Days required, median (range)
Spoligotyping	6 (1–26)
Culture confirmation	28 (6–64)
Susceptibility testing	37 (23–81)
RFLP typing	75 (24–160)

* RFLP, restriction fragment length polymorphism.

**Figure 1:**
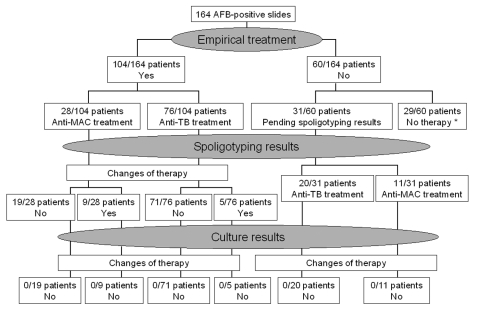
Clinical and therapeutic implications of spoligotyping results in treating suspected mycobacterial diseases. AFB, acid-fast bacilli; pts, patients; MAC, *Mycobacterium avium* complex; TB, tuberculosis. *Twenty-five patients did not begin treatment because they did not have clinical and radiologic features of tuberculosis. Four patients died within a few days after admission without receiving any antimycobacterial drug.

The use spoligotyping in determining treatment for 164 episodes of suspected mycobacteriosis was evaluated (Figure 1). In 25 episodes, patients did not begin antimycobacterial treatment because clinicians judged the AFB results to be not suggestive of true mycobacteriosis; none of these patients had clinical and radiologic features of tuberculosis (virtually all of these were infections with *M. gordonae* and *M. xenopi* isolates). Four patients died within a few days after admission without receiving any antimycobacterial drug.

Clinicians waited until spoligotyping results were obtained before choosing the appropriate therapy for 31 other patients. Subsequently, 20 patients began standard antituberculosis regimens (isoniazid, rifampin, pyrazinamide, and/or ethambutol), whereas the 11 other patients began treatment for infection with *M. avium* (clarithromycin, ethambutol, rifabutin, and/or ciprofloxacin). In all cases, the choice of the treatment based on spoligotyping was not changed after culture and susceptibility test results were obtained.

Empiric antimycobacterial therapy was given to the remaining 104 patients before spoligotyping results were obtained. Seventy-six of these patients received antituberculosis treatment against *M. tuberculosis* infections, and 28 received therapy for infection with *M. avium* based on clinical presentation. Therapy was subsequently modified as a result of the spoligotyping results in 14 of these 104 patients. In 5 patients in whom spoligotyping results were negative and subsequent cultures showed nontuberculous mycobacteria, antituberculosis treatment was replaced with treatment for infection with *M. avium*. In contrast, in 8 patients in whom spoligotyping results were positive and subsequent cultures were positive for *M. tuberculosis*, treatment for infection with *M. avium* was replaced by antituberculosis treatment. In 1 patient, the homology of the spoligotyping pattern with patterns of other patients included in our data bank demonstrated that the isolate originated from an outbreak caused by multidrug-resistant strains. Consequently, therapy was modified to include second-line antituberculosis drugs, which previous in vitro susceptibility data had shown were active against the specific strains. Subsequent in vitro susceptibility data confirmed the spoligotyping results. False-positive spoligotyping results in 9 patients did not result in errors in prescribing treatment. These patients continued ongoing treatment, primarily on the basis of previous spoligotyping results or because of a positive response to treatment.

### Analysis of Different Isolates from the Same Patient

We analyzed 34 spoligotyping-positive specimens from 6 patients with successive episodes of culture-confirmed tuberculosis (≥3 months apart). In these patients, spoligotyping was modified to distinguish relapses from new infections. After the samples were decoded, all episodes were classified as true relapses, and no new infections were detected. The banding patterns of the successive specimens matched those of their corresponding initial isolates. The subsequent RFLP results confirmed those obtained with spoligotyping.

### Genotyping

The reproducibility of spoligotyping was demonstrated by the identity of results obtained with clinical samples and corresponding cultures from different anatomic sites in the same episode (18 patients), and from episodes of recurrent tuberculosis in the same person (5 patients). Thirty-nine distinct spoligotyping patterns were observed; 55% of the specimens were grouped into 10 clusters, and the others had unique spoligotypes. Sixty-one different RFLP patterns were seen in 64 isolates. Of these 61 patterns, 3 were shared by 2 isolates, while the remaining 58 patterns (95%) were observed in only 1 isolate.

**Figure 2:**
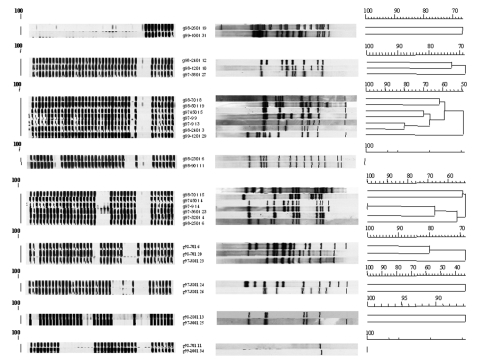
Spoligotype dendrograms generated by clustered *Mycobacterium tuberculosis* strains after computer analysis compared with the corresponding dendrograms of IS*6110* DNA fingerprints.

One of the 3 clusters identified by RFLP was a false cluster because it showed a 1-band pattern that correctly matched 2 different spoligotypes. In the remaining 2 RFLP clusters, complete concordance with spoligotyping was observed. The remaining 8 clusters detected by spoligotyping were not confirmed by RFLP analysis. Therefore, although it demonstrated 100% sensitivity, spoligotyping overestimated the number of clustered isolates by ≈50% (specificity 47.5%). Conversely, RFLP analysis had 100% specificity, but lower sensitivity. However, most of the isolates classified as clustered by spoligotyping but not by RFLP showed >50% similarity in their IS*6110* patterns (Figure 2). Using spoligotyping of clinical samples without culture confirmation, we were able to diagnose an *M. bovis* infection and rapidly identify 2 cases of recurrent tuberculosis in patients with the same spoligotyping pattern in both followup specimens and the initial *M. tuberculosis* strain isolated several months earlier.

## Discussion

Recent characterization of biologic markers for typing *M. tuberculosis* strains has greatly facilitated and improved tuberculosis epidemiology. IS*6110* RFLP typing is the most widely used method for differentiating *M. tuberculosis* strains (15–19). However, RFLP analysis requires growth of mycobacterial colonies, which involves a consistent lag time between epidemiologic events and typing results. Thus, time remains a limiting factor in obtaining a highly effective method for epidemiologic surveys and preventing the spread of disease.

The development of new PCR-based typing methods (4,5,9,10,20–23) has allowed rapid mycobacterial identification to be combined with epidemiologic typing results. Thus, molecular epidemiologic information can be combined in the context of epidemic events and tuberculosis transmission. Spoligotyping appears to have the specific characteristics needed to satisfy these issues of epidemics and tuberculosis transmission (24–28). This method permits the concomitant identification and differentiation of *M. tuberculosis* strains and avoids the timing problems associated with the slow growth of these bacteria (10,11). However, although spoligotyping can be used with clinical specimens (9), the usefulness of any PCR-based typing method in the clinical setting has yet to be demonstrated. In addition, although previous studies have investigated the effect of specific methodologic issues on the performance of several typing techniques (20–22,24–30), we have demonstrated the usefulness of a PCR–based technique in the clinical setting for diagnostic purposes, as well as for epidemiologic studies of tuberculosis transmission.

We have also shown that spoligotyping can be satisfactorily used with clinical samples. The performance of spoligotyping was satisfactory with all clinical specimens used, and its specificity and sensitivity were 98% and 96%, respectively. These features are comparable with those of commercial PCR methods used for detecting *M. tuberculosis* from clinical samples (19). Moreover, the opportunity to combine rapid diagnostic information and molecular epidemiologic data represents an important advance in the epidemiologic control of tuberculosis.

Several molecular typing studies have compared different methods for *M. tuberculosis* typing. IS*6110*-based RFLP has been found to be more discriminative than direct repeat–based spoligotyping (20–31). Although our data confirm that spoligotyping vastly overestimates the number of clustered isolates, this method has a lower discriminatory power than IS*6110*-RFLP. However, spoligotyping also has a higher negative predictive value, thus enabling the clinician to exclude a particular clustered strain as a cause of infection, if known drug-resistant variants are present.

This study showed that spoligotyping can provide useful data to clinicians in different settings. Although co-infection with *M. tuberculosis* and nontuberculous mycobacteria cannot be ruled out, the ability to differentiate between *M. tuberculosis* and other mycobacteria was demonstrated in 27% of the patients. Some patients began therapy after evaluation of spoligotyping results, while others changed treatments because spoligotyping did not confirm the initial diagnosis. Time of response for spoligotyping was shorter than that of culture confirmation of tuberculosis by a median of 20 days and that of susceptibility results by a median of 29 days. In addition, a median of 6 days was needed for obtaining spoligotyping results, compared with 75 days for RFLP typing results.

Comparison of molecular typing patterns identified 6 patients with reactivation of tuberculosis caused by the same strain of *M. tuberculosis*, a finding that can differentiate between relapse and new infection in a new episode of tuberculosis. In 1 patient, we found that the spoligotyping pattern was identical to that of other strains that belonged to a cluster of multidrug-resistant tuberculosis. This observation resulted in the modification of antituberculosis treatment 34 days before susceptibility data were available. Moreover, information obtained by spoligotyping was relevant and useful in therapeutic management of ≈33% of the patients.

The clinical utility of spoligotyping may not be fully apparent by analyzing the results of this study, primarily because of the lack of clustered episodes of tuberculosis during the study period. However, spoligotyping would have been useful during a period or in a setting characterized by the emergence of *M. tuberculosis* outbreaks (8,32,33). We have also shown that the usefulness of spoligotyping is increased when results are compared with data on other tuberculosis patients and a DNA database on *M. tuberculosis* strains.

In conclusion, this study underscores the need to implement rapid molecular epidemiologic methods in managing tuberculosis epidemics. We have shown that spoligotyping is a useful method for screening and epidemiologic control of tuberculosis dissemination, particularly when results are required quickly, such as in outbreaks, or in the management of transmission of multidrug-resistant tuberculosis, especially in restricted high-risk situations such as prisons, schools, and hospitals.
